# Major surgery delegation to mid-level health practitioners in Mozambique: health professionals' perceptions

**DOI:** 10.1186/1478-4491-5-27

**Published:** 2007-12-06

**Authors:** Amelia Cumbi, Caetano Pereira, Raimundo Malalane, Fernando Vaz, Colin McCord, Alberta Bacci, Staffan Bergström

**Affiliations:** 1 Independent public heath consultant, Maputo, Mozambique; 2 Division of International Health (IHCAR), Karolinska Institutet, Stockholm, Sweden; 3 Higher Institute of Health Sciences, Maputo, Mozambique; 4 School of Public Health, Columbia University, New York, USA; 5 World Health Organization, Copenhagen, Denmark

## Abstract

**Background:**

This study examines the opinions of health professionals about the capacity and performance of the 'técnico de cirurgia', a surgically trained assistant medical officer in the Mozambican health system. Particular attention is paid to the views of medical doctors and maternal and child health nurses.

**Methods:**

The results are derived from a qualitative study using both semi-structured interviews and group discussions. Health professionals (n = 71) were interviewed at both facility and system level. Eight group discussion sessions of about two hours each were run in eight rural hospitals with a total of 48 participants. Medical doctors and district officers were excluded from group discussion sessions due to their hierarchical position which could have prevented other workers from expressing opinions freely.

**Results:**

Health workers at all levels voiced satisfaction with the work of the "técnicos de cirurgia". They stressed the life-saving skills of these cadres, the advantages resulting from a reduction in the need for patient referrals and the considerable cost reduction for patients and their families. Important problems in the professional status and remuneration of "técnicos de cirurgia" were identified.

**Conclusion:**

This study, the first one to scrutinize the judgements and attitudes of health workers towards the "técnico de cirurgia", showed that, despite some shortcomings, this cadre is highly appreciated and that the health delivery system does not recognize and motivate them enough. The findings of this study can be used to direct efforts to improve motivation of health workers in general and of técnicos de cirurgia in particular.

## Background

In the aftermath of independence, building on experience in other countries, the Mozambican health system introduced new professional cadres to deliver basic comprehensive services, mainly in rural areas. Thousands of frontline health workers from basic to mid-level cadres were trained. The introduction of these cadres comprised the técnico de medicina, a mid-level medical practitioner, a key cadre at district level with clinical and managerial skills [1]. In line with this policy, a new cadre, 'técnicos de cirurgia' (TCs), able to perform emergency surgery, obstetrics and traumatology in the difficult conditions of rural hospitals, was introduced in 1984. At the time the need for these services was aggravated by emergencies created by a worsening civil war [2].

The TC in Mozambique does not have a medical degree; candidates are recruited mainly among the best mid-level medical practitioners or nurses, with substantial experience in rural areas. They undergo an intensive training programme, learning under the tight supervision of senior surgeons, comprising two years of training at Maputo Central Hospital and one year of internship in a provincial hospital [3]. The introduction of these cadres was met with some resistance from medical doctors and nurses. Among some of this staff, TCs were perceived as second class professionals leading to lack of consideration and commitment in the pursuit of their training. Nonetheless, TCs are usually assigned as the only 'surgeon' in a rural hospital with functioning theatre. All such health facilities in Mozambique are now staffed with at least one TC, the predominant cadre providing much needed emergency surgical care in rural areas. The young doctors deployed at this level have limited surgical experience and are in fact often being trained by this cadre of 'non-physicians' to perform major surgery.

Moreover, a recent study that compared the working histories of medical doctors and TCs shows that there is a very high degree of retention of TCs at the district hospital level, whereas almost all medical doctors posted there are gone within three years. Seven years after graduation more than 80% of TCs remain at district hospital level, whereas the corresponding percentage for medical doctors is zero [4]. Both this retention figure and the cost effectiveness data are strong arguments that for decades to come TCs will have a prominent place.

Today, despite interruptions in training and some losses from death and departure from government service, there are still 51 out of the 62 trained to date (2007) alive and practicing, mostly in rural areas.

The quality of their work has been shown to be very good [3,5], but there are still questions among professionals about their competence, and there are problems with morale among the TCs, relating principally to professional recognition and salary. Similar problems have been noted in other countries [6,7].

In the last few years of the HIV/AIDS epidemic, the growing awareness of the difficulties in retaining medical doctors in rural areas and the brain drain from low-income to high-income countries has renewed the interest in looking at alternatives of providing care. Recently, after the completion of this study in Mozambique, it was decided to give this category of mid-level health care provider recognition by additional training, leading to an academic degree.

Measures to address the challenge of the scarcity of human resources for health have been extensively revamped in recent years [8-10]. The Mozambican experience is paralleled by other countries [6], in which the delegation of major surgery to non-doctors is particularly substantial – notably in Tanzania [7] and in Malawi [11]. An assessment of the work performance of the TCs showed that more than 90% of all caesarean sections, obstetric hysterectomies and laparotomies for ectopic pregnancy are carried out by TCs [4]. A similar scenario has been found in our recent study at district level in Tanzania [3]. The same pattern emerges from our recent study in Malawi, which shows that about 90% of all caesarean sections at district hospital level are carried out by surgically trained clinical officers and with unexpectedly good results [12].

The present study is part of a broader ongoing evaluation, which assesses the use of TCs in providing basic surgery in rural areas, mainly the emergency obstetric care; the evaluation comprises four main studies (themes): (i) work performance of the TCs, (ii) comparison between the working histories of medical doctors and TCs, (iii) perceptions of the TCs about themselves and (iv) perceptions of other health professional about the TCs.

The relationship between the TC and other health professionals is important. Anecdotal information suggests that relational issues between TCs and other staff, mainly medical doctors affect motivation and performance of the TC. However, in Mozambique, no research has been conducted that portrays the views of health workers about the TC. Thus, the purpose of this study on health professionals' perceptions and the first of its kind, was to document the opinions and attitudes of various health professionals toward TCs, using approaches and methods previously reported [13-15].

The original term used in Mozambique ('técnico de cirurgia'; TC) is preferred in this exploratory study. Other titles are used in other countries as noted by Dovlo [16]. Expressions such as 'clinical officer', 'medical assistant', 'assistant medical officer' and 'health officer' mean different things in different countries.

## Methods

Mozambique, a long coastal country, has important developmental differences among the different regions; health resources as well as other resources are unevenly distributed benefiting the cities, particularly Maputo City, the national capital of the country. To take into account this range of differences, the study was conducted in three provinces, one in each of the three regions of the country: Nampula in the north, Zambézia in central Mozambique and Gaza in the south. In addition, two health facilities in the Maputo province were included. In Maputo city a number of hospital-based specialists were interviewed.

Moreover, the three provinces were chosen because they have the largest number of TCs and rural hospitals in their respective region. In each province, the interviews were conducted in all health facilities providing surgical care, yielding a total of 21 health units (two central hospitals, two provincial hospitals, two general hospitals, 12 rural hospitals and three health centres).

Health professionals were selected to capture a diversity of views: from health managers at system level to health care providers at the facility. During the pre-testing of instruments, it became clear that female participants (maternal and child health nurses) would not express freely their feelings in the group discussions. Furthermore, a certain reluctance to tackle openly the relationship issue was observed. Thus, the methods were adjusted to allow a better participation of these cadres and hence the individual interviews at facility level, initially planned only for medical doctors were expanded to include MCH nurses in the selected health units.

The study was conducted by a team of seven members: the three first authors and four provincial health workers from the evaluated provinces.

This exploratory study mainly examined the health workers' general opinions on the role played by the TC. In addition, the views of the health staff were assessed in other themes in order to explore and elicit reasons influencing the general opinion. Anecdotal information suggests that the perceived quality of care, performance, and relationships and collaboration with health facility team affect opinion and acceptability of health workers in regard to the TC. A fourth area included in the study was health workers' perceptions on the adequacy of support and supervision provided to TCs.

The study was carried out in the form of interviews using a semi-structured questionnaire with open questions in all institutions and health facilities; seventy-one staff were interviewed, comprising 18 general medical doctors, four gynaecologist-obstetricians, four orthopaedists, three surgeons, two public health specialists, 18 MCH nurses, nine operating room staff, eight district directors and five general nurses.

In addition, eight group discussion sessions of about two hours each were run in eight rural hospitals. Forty-eight participants attended the group discussion sessions. Medical doctors and district health officers were excluded in these discussions, because their hierarchical position could have limited free discussion.

Standard guidelines were developed for the group discussions and used in all the sessions held. The discussion began with a general question on the role played by TC. Towards the end of the session, the moderator probed for motivation, relationships, etc, if not already covered. During interviews and group discussions, notes were taken by both the main researcher and the assistant; immediately after the end of each session, data were compared for consistency and completeness and transcribed verbatim.

Interview data analysis comprised identifying and marking key points from each question (area of study) in each interview. Subsequently the emerging themes were identified and grouped by each health professional group. Focus group data were coded, analysed and summarised according to the different research topics.

Regarding core issues, no major differences emerged comparing interviews and group discussion data. However, the interview data were richer, thus selected interviewee responses translated verbatim from Portuguese to English are quoted in italics.

## Results

Medical doctors represent the largest (31) group of our interviewees. Among them about two thirds (19) are managers at provincial level (9) or medical officers/hospital directors at district level (11). Around one third are specialists, this group has a multifaceted relationship with the TC; their opinions, especially outside Maputo, are mainly those of carers of the patients referred by TCs, internship supervisors, and in some instances also colleagues. All interviewed health professionals were familiar with tasks carried out by the TC. Participants appear to have been open about their views during the group discussions but more frank about relationships during the interview. Overall, we focus on the interview method, limiting group discussion to general comments about the broad picture of the health professional views.

The findings give an overview of health staff in the four selected areas. Some emerged during data analysis, eliciting general opinions on the role played by the TC, the adequacy of their training, relationships with health facility team and career progression and remuneration.

In general, health staff is by and large positive to the TCs. In more than half the interviews and in the majority of the group discussions the opinions were predominantly positive. Nonetheless, in a limited proportion of interviews some negative aspects were pointed out. Criticism was more frequent among managers and specialists mainly those working outside Maputo.

### The role played by the "técnico de cirurgia"

Results from both interviews and group discussions show that the general opinion about the role played by TC is overwhelmingly positive. The interview data analysis identified seven themes most frequently mentioned in the interviews, which are summarized in Table 1. The questionnaire was open ended, the respondents referring to these different areas spontaneously.

**Table 1:** Health professionals' judgement of the role of TCs in various parameters.

	**Number interviewed**	**Their role is important**	**Role more important for maternal care**	**Provide life saving skills**	**Alleviate competition for scarce resources**	**Provide surgical emergency to a vast geographical area**	**Have a key role in rural hospitals**	**Replace the medical doctor (surgeon)**	**Contribute to surgical care provision at 3rd and 4th level**
**Medical Doctors**	**31**	**27**	**5**	**3**	**8**		**5**	**6**	**5**
Provincial Managers	7	7	2		3		1		3
District Level	12	12	1	2	4		3	3	1
Specialists in central & prov. hosp.	9	4	1	1	1		1		1
Specialists in Maputo City	2	2	1					1	
Specialists at Maputo Central Hosp.	2	2						2	
**MCH Nurses**	**18**	**16**	**7**	**6**	**3**			**3**	
District Level	15	13	4	6	3	4		2	
Provincial Managers	3	3	3					1	
**Chief nurses**	**5**	**4**		**1**	**1**				**1**
**Theatre room team**	**9**	**9**	**1**	**3**	**3**	**4**	**2**		
**District health directors**	**8**	**8**	**4**	**2**	**1**	**3**	**2**		
Total	**71**	**64**	**17**	**15**	**16**	**11**	**9**	**9**	**6**

Note: Given the multiple answers per interviewee, the total answers add more than the number of interviewees.

As illustrated in Table 1, the vast majority, 64/71 (90%), of interviewees considered TCs to be important. Among medical doctors at all levels, bar the specialists working outside Maputo, this figure reached 100%. Other health staff interviewed at district level had a similar opinion; 37/40 (90%) considering TCs to have an important role. Interviewees, mainly non-physician staff, mostly associate the TCs' importance with the key role they play in maternal care and life saving skills in general. Besides this, the general opinion was that Rural Hospitals are almost completely dependent on TCs for surgical activity, for which they have adequate and usually appropriate training.

*"It is like this, the TCs are very important for the life of our health units: first we don't have specialists to address the country's needs (...) any health unit without a TC suffers a lot due to the lack of this cadre. The work that they carry out, I am not going to say perfect but it is very good. ... We, the medical doctors, have a very limited training ... beside that I am not interested in surgery and obstetrics." *(Medical doctor, district hospital director)

Besides, it was noted during this study that the levels of absenteeism are lower than that of medical doctors.

Interviewees across all health professional groups also associate the presence of a TC in a district with an important reduction of costs. The surgical activities performed by the TCs lessen the pressure on the meagre healthcare resources by reducing the number of patient referrals. They reduce both emotional and financial costs for the patients and their families:

*"He [the TC] is very important; in the past, due to the lack of this cadre, there were many problems; we had to refer everything to another district and the provincial hospital in another province. The disruptions caused by this were a real problem, either in money spent for fuel or for the ensuing costs to the patients. Mainly for us here, in district of ..., the district of ... and provincial hospitals are too far from here for us to refer patients there. But now it is possible to manage [emergencies] locally, it's easy to treat the patients here." *(Chief Nurse in charge of nursing care, district hospital)

*"...when the TC is absent the result is catastrophic; many resources are spent for [patient] referrals, transport, etc"*. (Medical Doctor, District Clinical Officer)

In addition, some health workers at district level associate the responsibility of TCs not only with the hospital where they are deployed but for a larger geographical area:

*"... In this region it is a very important work because he is the only one, it is a rural hospital that serves three districts. He has been saving many people"*. (MCH nurse, district hospital)

*"I think that [the TC] is very important, since this is a rural hospital, thus a referral health facility. There are many inhabitants and the medical doctor does not have sufficient training in surgery." *(Medical doctor, rural hospital)

Interviewed health professionals, mainly the medical doctors at provincial level, judged that the work of the TCs also has a positive impact on the surgical care provided at levels above the rural hospital, either directly or indirectly. They pointed out that although the TCs were envisaged to provide surgical care in rural hospitals, a noticeable proportion of TCs are deployed at provincial and central hospitals, that the TC's work at district level greatly alleviates the pressure and workload of second and third referrals units:

*"Well, our TC is good, because without him I don't know what would be in terms of the rural hospital [where] he is the surgeon; here in the provincial hospital he works in shifts in equal terms with the other specialists [surgeon, obstetrician and orthopaedic]; when one specialist goes on vacation, she/he is replaced by the TC. At rural hospital level they [TCs] provide all [types of] care and they decrease the provincial hospital workload, [can you] imagine without their presence [in the districts], what would be the workload at the provincial hospital?" *(Medical doctor, provincial health authority)

### Training and quality of care

When questioned about the perceived quality of care/performance of the TCs, more than half of the interviewed health professionals – but very few group discussion participants – addressed the issue by talking about the TC training. Selected sub-themes that emerged from interview data analysis are presented below. The overall opinion, mainly of the medical doctors (10/12) at district level, was that TCs are adequately trained:

*"The TC is well trained. I wouldn't change anything in his training. I'm speaking about the specific situation here ..." *(Medical doctor, rural hospital director)

Nonetheless, some shortcomings were pointed out in the discussions held. A number of interviewed medical doctors spontaneously brought up issues they felt a need to be looked at, such as: theoretical and clinical skills, the internship process and its organization, limited orthopaedic capacity, and the need for a clear definition of the level/limit of intervention by these cadres.

### Surgical, theoretical and clinical skills

Health professionals, mainly medical doctors, consider that the TCs have good surgical skills, mainly to tackle obstetric emergencies. A few specialists felt that orthopaedics should be strengthened although acknowledging that the available training time is an important limitation.

Some interviewed medical doctors considered the TCs' pharmacological knowledge and prescribing competence insufficient. Few doctors suggested that TCs with a background training as general nurses or nurse specialists have more limitations in clinical skills than the TCs entering with a background as "técnico de medicina" – a mid-level cadre with three year's training in clinical skills (diagnosis and treatment).

One medical doctor raised the critical issue of neonatal care:

*"In the district of ... we have two TCs; [before] we had [also] an expatriate gynaecologist. The TC had better surgical skills and the gynaecologist recognized this [fact]. In relation to quality and capacity of surgical interventions, they are good. I have reservations on their pre- and postoperative abilities." *(Medical doctor, public health specialist & provincial director)

*"...the only thing is that they use a lot of antibiotics and expensive ones; all the caesarean sections are treated with antibiotics; all the equipment is sterilized in the theatre room and it is the surgery team who controls..." *(Medical doctor, district medical officer)

### Internship

In general, the interviewed specialists/consultants judged that trainees during their internship at Maputo Central Hospital are not adequately supervised. One surgeon added that in his opinion, these cadres should have a longer period of internship at provincial level, having conditions similar to the ones waiting for the TC once in a rural setting. However, this surgeon and some other specialists considered that the process and organization of the internship at provincial hospitals needed to be strengthened to serve adequately this end. Besides the problems with provincial hospital capacity itself, two specialists outside Maputo noted that the informal approach followed negatively affects the organization of the internship:

*"... The TC should be trained in a provincial hospital and spend more time at a provincial hospital and less in the Maputo Central Hospital: (a) until their arrival at the Provincial Hospital for their internship they don't have sufficient [practical skills]; in Maputo Central Hospital there are numerous students ... and they all stay behind one consultant, thus in Maputo Central Hospital the TC has less supervision, which means fewer opportunities to practice. (b) The provincial hospital is the internship field nearer to and similar to the conditions where the TC is going to work*."... (Medical doctor, expatriate obstetrician-gynaecologist)

In order to further improve the performance of these cadres, some interviewees drew attention to the above shortcomings and thought they should be addressed, either during the pre-service training of these cadres, or through a well designed hands-on, on-the-job training programme, e.g. by The National Surgery Programme, organizing regional training courses for these cadres once a year. However, for a number of reasons, not all the TCs have been able to attend these courses. The professionals interviewed felt that the training approach of these courses needs to be modified thoroughly; the specialists working in the referral hospitals with major contact with the TCs should have an active role in this training programme with emphasis on a more practical approach, implying a hands-on and problem-specific training process:

*"...An in-service training is necessary because in the districts they have to take care of all areas – obstetrics, gynaecology, orthopaedics and surgery; in order to further improve their performance in other areas, they could stay [return] for a week in a provincial hospital and besides [general] surgery they could also see [be trained] obstetrics, orthopaedics. Or any other type of training to prepare them because they work alone in the districts in remote areas. ..." *(Chief Nurse, in-charge of nursing care, district hospital)

A small group of professionals, mainly specialists, raised concerns regarding practice regulation; they considered that in some instances TCs intervene above their abilities:

*"There should be a regulation regarding the interventions that the TCs can perform; some perform surgery above their capacities, for example: fistulas, prostate cancer, etc. There should be a regulation of what they can do"*. (Medical doctor, provincial health authority)

### Relationships and collaboration

In the group discussion, notwithstanding probing efforts, very few participants (8/48) addressed the issue and five of them stated that 'there is good collaboration'. Although individual interviewees were more open and frank, only just above half of the interviewed health professionals addressed this issue. The majority of them referred to a variety of difficulties in collaborating with TCs. In particular, their interactions with medical doctors at district level have been considered problematic. Interviewees of different categories felt that the skills of the TC represented a threat to the power of the medical doctor and the district officer, resulting in conflicting relationships:

*"We, the medical doctors, don't have knowledge of surgery and they try to show this; that they are on top [more skilled than us] and this creates conflicts with the medical doctors. Sometimes there are many conflicts. During the training itself, they should know that in spite of their surgical skills, they are technicians [mid-level cadre] ... and that they are subordinated to the medical doctor and that they are going to work with a team"*. (Medical doctor, provincial medical officer)

*"They have more value because they are considered Kings in the district; the TC performs surgical interventions and the medical doctor writes out prescriptions of paracetamol [tablets] ... this creates conflicts with medical doctor since s/he doesn't have enough surgical skills such as caesarean section, ... ... and a lot more. In fact, you will see that the medical doctor opinion will be different from mine"*. (Expatriate surgeon Maputo City)

Some TCs are considered arrogant. Some health professionals referred to a lack of openness from the TCs, which limited collaboration with other colleagues. This attitude sometimes is a source of problematic relationships and it hinders the learning process of other cadres and maintains levels of high workload for the TC. Some interviewees noted that the recently-launched training programme on safe motherhood trained medical doctors and mother and child health nurses; but due to the lack of collaboration of some TC in some districts these trained cadres are not applying the new skills acquired:

*"...There is no space ... the medical doctors who went to safe motherhood training programme for obstetric care do not make use of this training, due to lack of collaboration with the TC. As a result TCs continue with a high workload and the participation of medical doctors in the implementation of the safe motherhood strategy is limited"*. (District health director)

*"... They need clear information because often the TC thinks that he is alone ... another thing is the training, I've seen that he wouldn't let the MCH nurses perform aspirations of abortions. If he thinks they don't have the skill, he should train them; it would be a way of alleviating his workload. It is a waste because the MCH nurses have had the preparation in the safe motherhood training programme and in the District of ... they are not using it [the skills acquired]. It has to do with the TC himself and the time they stay in the same district, if they stay for three to five years they end up becoming the owners of everything. ... The medical doctors, who attended the safe motherhood training programme, once back to their districts; often do not have the chance to perform. Meanwhile, the TC continues with high workload." *(MCH nurse, in-charge of provincial mother and child health care)

Two provincial directors suggested that character problems among TCs as well as among medical doctors are important in the existing relationship between the two categories.

*"... Something very important is missing in the TC profile and it is the training itself that is failing, he [TC] has to understand that he is not the king... on the other side, medical doctors are trained in an atmosphere of vanity..." *(Medical doctor, Provincial Director)

However, some interviewees acknowledged a good relationship with TCs:

*"I have good relationship with him; he is indefatigable ... if all TCs were like him the country wouldn't have problems. ... The problematic relationship depends on the medical doctor who is there; hardly the medical doctor and the TC sit at the same table. ... The existing relationship have to do with the personal temperament... it's bad, the war weakens the authority. ... My congratulations to him I only pray [hope] that the medical doctor coming to replace me will work well with him." *(Medical doctor, rural hospital director)

### Career progression and remuneration

When questioned about the adequacy of support and supervision provided to the TC, most interviewees and group discussion participants raised issues about insufficient incentives, inadequate working conditions, high workload, insufficient recognition/valorisation and only a few, mainly medical doctors interviewed raised the career progression and remuneration issue. However, this last issue appeared to be more important and was thus selected. All staff addressing this issue judged the TCs' career perspective as inadequate. They think that TCs should not be considered mid-level cadres, since they have more years of training, far heavier responsibilities, unique skills at district level and a higher workload than most mid-level staff. The salary issue was more controversial, with diverging views among health professionals. Although the overall view is that the TC pay level is low, some interviewees affirmed that the TCs salary problem is just the same as all health workers'. Other sources of income and/or incentives were mentioned during the discussions, but interviewees judged them insufficient. They comprise housing, transport, private practice, etc, and they are mostly dependent on local initiatives. However, some interviewees stressed the inadequacy of the career pathway and remuneration:

*"... An individual spends six years in school and continues to be considered mid-level [it's unjust]... There is a huge gap between the salaries of medical doctors and the TCs ... even a newly-trained medical doctor earns more than four times the TC's salary. It's not a designation problem but a problem of career qualification. It's necessary to distinguish the areas, not all [workers] are equal, and a nurse has three training years less than the TC. The TCs are being damaged in relation to wages"*. (Medical doctor, specialist, Maputo)

Some interviewees, mainly medical doctors and MCH nurses, considered the TCs to be the most disadvantaged health professionals partly due to career definition problems. Thus, they found the payment of this cadre very low in absolute terms as well as when compared with other professionals within the health system and outside it. Moreover, a few of the interviewees considered that the salary level affects the TC morale and motivation with ensuing behavioural problems. In few interviews illicit charges were also mentioned:

*"... also the income is insufficient, because, sometimes we give a glance [at the salaries] and there is no difference between them and other mid-level cadres ... the salary is very low. They work a lot, that it is why sometimes they find themselves obliged to ask for illicit charges. When someone comes and asks for an abortion we send her to them. There are persons who ask for [abortions] and then they speak out outside that they were charged whilst it is they who looked for [asked for] it. Therefore, at least their salary should be increased." *(MCH nurse, in charge of the district mother and child health care)

## Discussion

The views portrayed in this study hardly include the opinions of managers at central level; this notwithstanding efforts made to interview professionals in the Ministry of Health. The assistants who helped in note-taking were not always trained for the task, as in each province a provincial manager filled this 'position'. Besides the lack of training he or she was always known to the interviewees and to the group discussion participants; their colleague from the provincial authorities, thus in some stances was in higher hierarchal position. All this, may have affected the quality of data collected through some of the interviews. In spite of these limitations, the information obtained appears to provide an adequate picture of the health workers' feelings towards the TCs. The same questionnaire was administered across all different health professional groups and this enhanced comparability of answers. The themes that arose were consistent across interviews in the different provinces and different categories of health professionals.

This study shows that TCs in general are appreciated by other categories of health staff and that their contribution to surgical care is considered important by more than 90% of physicians and other staff. It is almost a universal opinion of the interviewees that the TCs are critical for surgical emergency care delivery, particularly in rural areas. This view has been pointed out in other studies on TCs in Mozambique [17]. Interviewees and participants in group discussions placed great emphasis on the life-saving skills of these cadres and considered that TCs have a key role in the rural hospitals, which serve vast geographical areas. They contribute to cost reduction and their activities alleviate the workload of the provincial hospitals.

The appreciation from health workers that TCs contribute significantly to a cost reduction has been confirmed in a recent study, in which it was established that the cost-effectiveness of TCs in relation to medical doctors as far as caesarean section is concerned is approximately three times more favourable for TCs than for medical doctors. Even if the salary of TCs were doubled, this ratio would be 2.5 times more favourable [18].

Whilst rural hospitals in Mozambique play a key role in providing emergency surgical care, they are very few, only 32 in 2002. Thus, they offer surgical referral care to a cluster of districts, from three to five. Consequently, these hospitals serve as first surgical referral units for vast geographical areas which means long distances of up to 300 Km of frequently bad roads and serving large populations (from 90,000 to 1,500,000 inhabitants) and a considerable number of health facilities. Some of the interviewees, mainly mid-level cadres at district level, highlighted this fact as it greatly amplifies the importance of the role played by the TC.

In the initial decade of the training of TCs in Mozambique there was a clear opinion, above all among senior surgeons, that the introduction of TCs was only acceptable as a temporary solution to a critical problem of scarcity of human resources for health. Thus, no due attention was paid to the institutional and organizational implications of introducing a cadre playing such an important role. As a result the career progression of these cadres and other PHC practitioners is ill-defined. Some interviewed health professionals, mostly the medical doctors, stressed the problems of career progression and low pay of TCs, which to a certain extent leads to low motivation among the TCs.

Although in the interviews and group discussions the overwhelming majority of health professionals acknowledged the major role played by these cadres in the provision of surgical care, recognition is in fact inadequate. TCs hold unique and vital skills at district level. However, they are still considered and paid as mid-level cadres. They play a marginal role within the district management structure. These issues compounded by the elitist culture of the medical doctors, are important in shaping the existing relationships among them and other health professionals.

The findings of this study can be used to direct efforts to improve motivation of health workers in general and of TCs in particular.

## Conclusion

Health professionals were almost always positive about the work carried out by TCs, though, in some instances, their pre-service training in therapeutic (pharmacologic) management was considered insufficient. Several interviewees considered that a more practical training should be decentralized to provincial hospitals, having a workload situation closer to the district level than hospitals in Maputo, the national capital. The TCs' professional status was not considered commensurate with the job they are asked to do, and career and remuneration issues continue to be unsolved problems. It was often recognized that TCs contribute to lowering costs by avoiding otherwise unnecessary referrals from district to provincial level for surgical and obstetrical emergencies requiring major surgery. The sustainability issue was raised frequently and health workers generally recognized that the retention of TCs at district level was much higher than that of doctors, and that without TCs it will be impossible to provide surgical services in rural areas for decades to come.

## Competing interests

The author(s) declare that they have no competing interests.

## Authors' contributions

AC designed the study and performed the interviews assisted by CP and RM, who prepared the localization of interviewees and organized the field work. FV, CM, AB and SB contributed with background documentation and with critical views on design and implementation of project. They also collaborated actively with AC and CP in analyzing all data collected and in elaborating the manuscript.
